# A Lipidomic Perspective of the Action of Group IIA Secreted Phospholipase A_2_ on Human Monocytes: Lipid Droplet Biogenesis and Activation of Cytosolic Phospholipase A_2_α

**DOI:** 10.3390/biom10060891

**Published:** 2020-06-10

**Authors:** Juan P. Rodríguez, Elbio Leiguez, Carlos Guijas, Bruno Lomonte, José M. Gutiérrez, Catarina Teixeira, María A. Balboa, Jesús Balsinde

**Affiliations:** 1 Instituto de Biología y Genética Molecular, Consejo Superior de Investigaciones Científicas (CSIC), Universidad de Valladolid, 47003 Valladolid, Spain; rodriguezcasco@me.com (J.P.R.); elbio.leiguez@butantan.gov.br (E.L.); cguijas@ibgm.uva.es (C.G.); mbalboa@ibgm.uva.es (M.A.B.); 2 Laboratorio de Investigaciones Bioquímicas de la Facultad de Medicina (LIBIM), Instituto de Química Básica y Aplicada del Nordeste Argentino (IQUIBA-NEA), Universidad Nacional del Nordeste, Consejo Nacional de Investigaciones Científicas y Técnicas (UNNE-CONICET), Corrientes 3400, Argentina; 3 Laboratorio de Farmacologia, Instituto Butantan, Sao Paulo 01000, Brazil; catarina.teixeira@butantan.gov.br; 4 Centro de Investigación Biomédica en Red de Diabetes y Enfermedades Metabólicas Asociadas (CIBERDEM), 28029 Madrid, Spain; 5 Instituto Clodomiro Picado, Facultad de Microbiología, Universidad de Costa Rica, San José 11501–2060, Costa Rica; bruno.lomonte@ucr.ac.cr (B.L.); jose.gutierrez@ucr.ac.cr (J.M.G.)

**Keywords:** phospholipase A_2_, lipidomics, mass spectrometry, lipid signaling, inflammation, monocytes/macrophages

## Abstract

Phospholipase A_2_s constitute a wide group of lipid-modifying enzymes which display a variety of functions in innate immune responses. In this work, we utilized mass spectrometry-based lipidomic approaches to investigate the action of Asp-49 Ca^2+^-dependent secreted phospholipase A_2_ (sPLA_2_) (MT-III) and Lys-49 sPLA2 (MT-II), two group IIA phospholipase A_2_s isolated from the venom of the snake *Bothrops asper*, on human peripheral blood monocytes. MT-III is catalytically active, whereas MT-II lacks enzyme activity. A large decrease in the fatty acid content of membrane phospholipids was detected in MT III-treated monocytes. The significant diminution of the cellular content of phospholipid-bound arachidonic acid seemed to be mediated, in part, by the activation of the endogenous group IVA cytosolic phospholipase A_2_α. MT-III triggered the formation of triacylglycerol and cholesterol enriched in palmitic, stearic, and oleic acids, but not arachidonic acid, along with an increase in lipid droplet synthesis. Additionally, it was shown that the increased availability of arachidonic acid arising from phospholipid hydrolysis promoted abundant eicosanoid synthesis. The inactive form, MT-II, failed to produce any of the effects described above. These studies provide a complete lipidomic characterization of the monocyte response to snake venom group IIA phospholipase A_2_, and reveal significant connections among lipid droplet biogenesis, cell signaling and biochemical pathways that contribute to initiating the inflammatory response.

## 1. Introduction

The phospholipase A_2_ (PLA_2_) superfamily consists of a broad range of enzymes defined by their ability to catalyze the hydrolysis of the ester bond at the sn-2 position of glycerophospholipids. The hydrolysis products of this reaction, free fatty acid and lysophospholipid, serve as precursors for a variety of bioactive lipid mediators with important biological roles [1]. The PLA_2_s are systematically classified according to sequence homology criteria, and include 16 groups (I-XVI), most of them with several subgroups, comprising more than 30 proteins [1]. An alternative classification also exists that groups these enzymes into six major classes on the basis of biochemical similarities and/or cell regulation properties. These are the Ca^2+^-dependent cytosolic PLA_2_s, the Ca^2+^-dependent secreted PLA_2_s (sPLA_2_), the Ca^2+^-independent cytosolic PLA_2_s, the platelet-activating factor acetyl hydrolases, the lysosomal PLA_2_, and the adipose-specific PLA_2_ [2].

The sPLA_2_ family represents the largest class of PLA_2_ enzymes and possesses, as a common motif, a conserved His-Asp catalytic dyad [3]. sPLA_2_s are widely distributed in pancreatic secretions, inflammatory exudates, and also in arthropod and snake venoms. A variety of biological activities have been described for sPLA_2_s, including digestive actions, toxic activities (neurotoxic, myotoxic, hypotensive, etc.) and immune roles. In this regard, group IIA sPLA_2_ was defined as a pro-inflammatory PLA_2_, since its gene induction and synthesis were observed after cell stimulation by endotoxin and cytokines [3,4,5]. In contrast, another member of the family, the group V enzyme, is described as anti-inflammatory in some models [6,7,8].

sPLA_2_s have been often observed to cooperate with other PLA_2_s in eliciting certain biological responses. A prominent example of this is the mobilization of arachidonic acid (AA) and attendant eicosanoid production by innate immune cells responding to inflammatory stimuli [9,10,11,12]. The Ca^2+^-dependent cytosolic group IVA PLA_2_ (cPLA_2_α) is the essential enzyme in this process [12,13,14]. Depending on cell type and stimulation conditions, regulatory crosstalk mechanisms exist between cPLA_2_α and other sPLA_2_ enzymes present in the cells—in particular, those belonging to groups IIA, V and X—which results in the amplification of the AA mobilization response [15,16,17,18,19,20].

Previous work from our laboratory has utilized advanced mass spectrometry approaches to characterize multiple aspects of PLA_2_-mediated phospholipid fatty acid remodeling in cells of the innate immune system such as monocytes and macrophages [21,22,23,24,25,26,27,28]. In these studies, the activation mechanisms of multiple PLA_2_ enzymes expressed by the cells were characterized [21,22,23,24,25,26,27,28]. However, no approaches were undertaken to characterize the cellular responses to exogenously added sPLA_2_ enzymes. It has been suggested that the response of cells exposed to exogenous sPLA_2_ is dependent upon the nature of the lipid mediator generated on the membrane where the sPLA_2_ acts [29]. However, it is also known that some sPLA_2_s lack catalytic activity but still exert potent biological actions [30]. This has led to the proposal that some sPLA_2_ effects depend on protein–protein or protein–glycan interactions [3,31]. Once bound to its target(s) on the membrane, sPLA_2_s may exert their actions via activity-independent mechanisms that affect cellular functions or trigger a cellular response [3,31,32]. Additionally, the existence of a sPLA_2_ receptor for these enzymes has long been proposed [33]. Whether activity-based effects prevail over activity-independent effects, or both occur simultaneously, remains unclear.

Exposure of immune cells to exogenous sPLA_2_ occurs in numerous pathological situations such as inflammatory syndromes, sepsis, autoimmune diseases, and even bite or sting envenomations [3,4,5]. In this work, we have used advanced mass spectrometry-based lipidomics to analyze the effect of two different group IIA sPLA_2_, with and without catalytic activity, on human monocytes. The sPLA_2_ enzymes utilized in this study, termed Asp-49 sPLA_2_ (MT-III) and Lys-49 sPLA_2_ (MT-II), were purified from the venom of the Central American snake *Bothrops asper*. MT-III is a catalytically active enzyme similar to human synovial group IIA PLA_2_. MT-II is identical to MT-III, except for the replacement of Asp49 with Lys49 within the active site, which renders it catalytically inactive [34,35,36,37,38]. The combined use of these two PLA_2_s thus constitutes an excellent tool to distinguish between the activity-dependent and -independent actions of sPLA_2_ enzymes. Consistent with the previously described proinflammatory properties of MT-III, our data show that it promotes remarkable changes in the lipid composition of cell membranes, triggers lipid droplet biogenesis, and induces eicosanoid synthesis. None of these actions are induced by the inactive form MT-II. These data agree with previous work demonstrating that MT-III, but not MT-II induces phospholipid hydrolysis in murine muscle cells [38].

## 2. Materials and Methods 

### 2.1. Enzymes

Asp-49 sPLA_2_ (MT-III) and Lys-49 sPLA_2_ (MT-II) from Bothrops asper venom were purified by ion-exchange chromatography on CM-Sephadex C-50, using a KCl gradient from 0 to 0.75 M [36]. The complete amino acid sequence and toxicological profile of these enzymes have been previously described in detail [37]. The absence of endotoxin contamination in the batches used was demonstrated by performing the quantitative Limulus amebocyte lysate assay [39], which revealed no detectable levels of endotoxin (< 0.125 EU/mL).

### 2.2. Cell Culture

Human monocytes were isolated from buffy coats of healthy volunteer donors obtained from the Centro de Hemoterapia y Hemodonación de Castilla y León (Valladolid, Spain). Written informed consent was obtained from each donor. Briefly, blood cells were diluted 1:1 with phosphate-buffered saline, layered over a cushion of Ficoll-Paque, and centrifuged at 750 g for 30 min. The mononuclear cellular layer was recovered and washed three times, resuspended in RPMI 1640 medium supplemented with 40 μg/mL gentamicin, and allowed to adhere in sterile dishes for 2 h at 37 °C in a humidified atmosphere of CO_2_/air (1:19). Nonadherent cells were removed by washing extensively with phosphate-buffered saline, and the remaining attached monocytes were used the following day [40,41]. For experiments, subconfluent cell monolayers were incubated with serum-free medium for 1 h before the addition of sPLA_2_. After stimulation, the monocyte monolayers were washed twice with phosphate-buffered saline, scraped with a cell scraper, sonicated with a tip homogenizer twice for 15 s, and prepared for their further analysis by mass spectrometry, as described below. For eicosanoid determinations, supernatants were collected and prepared for mass spectrometry analysis as described below.

### 2.3. Cellular Staining and Fluorescence Microscopy

For these experiments, the cells were plated on coverslips on the bottom of 6-well dishes in a volume of 2 mL. The cells were fixed with 1 mL of 4% paraformaldehyde in phosphate-buffered saline containing 3% sucrose for 20 min. Afterward, paraformaldehyde was removed by washing the cells three times with phosphate-buffered saline, and Nile Red and 4′,6′-diamidino-2-phenylindole (DAPI) stainings were carried out by treating cells with these dyes at concentrations of 5 µg/mL and 1 µg/mL, respectively, in phosphate-buffered saline for 10 min. Coverslips were mounted on microscopy slides with 25 µL of a polyvinyl alcohol solution until analysis by fluorescence microscopy. Fluorescence was monitored by microscopy using a NIKON Eclipse 90i device equipped with a CCD camera (model DS-Ri1; Nikon, Tokyo, Japan). A mercury HBO excitation lamp (Osram, Munich, Germany) was used, and the fluorescence was recovered using the combination of a UV-2A (Ex 330–380; DM 400; BA 420) and a B-2A (Ex 450–490; DM 505; BA 520) filter, respectively. Images were analyzed with the software NIS-Elements (Nikon). Red and blue channels were merged with the Image-J software (version 1.52a).

### 2.4. Gas Chromatography/Mass Spectrometry (GC/MS) Analysis of Fatty Acid Methyl Esters

Total lipids from approximately 10^7^ cells were extracted according to Bligh and Dyer [42]. For separation of total phospholipids from neutral lipids, the following internal standards were added: 10 nmol of 1,2-diheptadecanoyl-sn-glycero-3-phosphocholine, 10 nmol of 1,2,3-trihepta-decanoylglycerol, 20 nmol of nonadecanoic acid, and 30 nmol of cholesteryl tridecanoate. Phospholipids were separated from neutral lipids by thin-layer chromatography, using *n*-hexane/diethyl ether/acetic acid (70:30:1, *v*/*v*/*v*) as the mobile phase [43]. For separation of phospholipid classes, the following internal standards were added: 20 pmol each of 1,2-diheptadecanoyl-sn-glycero-3-phosphoethanolamine, 1,2-diheptadecanoyl-sn-glycero-3-phospho-choline, and 1,2-dinonadecanoyl-sn-glycero-phosphoinositol. Phospholipids were separated twice with chloroform/methanol/28% (*w*/*w*) ammonium hydroxide (60:37.5:4, *v*/*v*/*v*) as the mobile phase, using plates impregnated with boric acid [44]. The bands corresponding to the different lipid classes were scraped from the plate, and fatty acid methyl esters were obtained from the various lipid fractions by transmethylation with 0.5 M KOH in methanol for 60 min at 37 °C [45,46,47,48,49]. Analyses were carried out using an Agilent 7890A gas chromatograph coupled to an Agilent 5975C mass-selective detector operated in an electron impact mode (EI, 70 eV). (Agilent Technologies, Santa Clara, CA, USA). Data acquisition was carried out both in scan and selected ion monitoring mode. Scan mode was used for compound identification, comparing with authentic fatty acid methyl ester standards, and the National Institute of Standards and Technology MS library spectra. Selected ion monitoring mode was used for quantitation, using 74 and 87 fragments for saturated, 83 for monounsaturated, 67 and 81 for diunsaturated, and 79 and 91 for polyunsaturated fatty acid methyl esters. A 37-component mixture (Supelco, Sigma-Aldrich, Madrid, Spain) was used for calibration curves.

### 2.5. Mass Spectrometry Analysis of Free Fatty Acids

The thin layer chromatography spots corresponding to the non-esterified free fatty acid fraction were scraped, redissolved in *n*-hexane, and analyzed separately in an Agilent 1260 Infinity high-performance liquid chromatograph coupled to an API2000 triple quadrupole mass spectrometer (Applied Biosystems, Carlsbad, CA, USA). The column was a Supelcosil LC-8 (150 × 3 mm, 3 μm particle size), protected with a Supelguard LC-8 (20 × 3 mm) guard cartridge (Sigma-Aldrich). The mobile phase was used on a gradient of solvent A (methanol with 0.01% ammonium hydroxide) and solvent B (water with 0.01% ammonium hydroxide). The gradient was started at 60% solvent A and 40% solvent B. The former was linearly increased to 95% at 10 min, and held at 95% solvent A until 18 min. The initial solvent mixture (60%, 40%B) was recovered at 20 min and the column was re-equilibrated for an additional 5 min before the injection of next sample. The flow rate was fixed at 400 μL/min. Non-esterified fatty acid fraction, extracted from silica plates and filtered, was re-dissolved in 100 μL of methanol/water 60:40 *v*/*v* and 90 μL were injected into the high-performance liquid chromatograph. The parameters for electrospray ionization source of mass spectrometer were set as follows: Ion spray voltage, −4500 V; CUR, 20 psi; GS1, 40 psi; GS2, 80 psi; TEM, 525 °C. The analyzer mode was set to Q1MS (DP, −70 V; EP, −10 V; FP, −300 V) performing a m/z scan between 100 and 400 with a step size of 0.1 amu. Non-esterified fatty acids were detected as [M − H]^−^ ions using the Analyst 1.5.2 software version (Applied Biosystems, Carlsbad, CA, USA), and chromatographic peaks were quantified by comparison with peaks of authentic analytical standards.

### 2.6. Liquid Chromatography/Mass Spectrometry (LC/MS) Analyses of Phospholipids

This was carried out exactly as described elsewhere [21,22,23,24,25,26,50,51], using a high-performance liquid chromatograph equipped with a binary pump Hitachi LaChrom Elite L-2130 and a Hitachi Autosampler L-2200 (Merck), coupled on-line to a Bruker esquire6000 ion-trap mass spectrometer (Bruker Daltonics, Bremen, Germany). Ethanolamine-containing phospholipids (PE) and phosphatidylinositol (PI) species were detected in negative ion mode as [M − H]^−^ ions in MS experiments. Choline-containing phospholipids (PC) species were detected in positive ion mode, as [M + H]^+^ ions by MS. Acyl chains in PI and PE species were identified by multiple reaction monitoring MS^2^ experiments on chromatographic effluent by comparison to previously published data [21,22,23,24,25,26,50,51]. For the identification of acyl chains in PC species, ionization was carried out in negative mode with the post-column addition of acetic acid at a flow rate of 100 mL/h as [M + CH_3_CO_2_]^−^ adducts, and acyl chains were identified by MS^3^ experiments. Quantification was carried out by integrating the chromatographic peaks of the previously identified phospholipid species and comparing with an external calibration curve made with authentic standards.

### 2.7. Liquid Chromatography/Mass Spectrometry (LC/MS) Analyses of Eicosanoids

Analysis of eicosanoids by LC/MS was carried out exactly as described elsewhere [6,25,52], using an Agilent 1260 Infinity high-performance liquid chromatograph coupled to an API2000 triple quadrupole mass spectrometer (Applied Biosystems, Carlsbad, CA, USA). Quantification was carried out by integrating the chromatographic peaks of each species and by comparing with an external calibration curve made with analytical standards [6,25,52].

### 2.8. Immunoblot

Cells were lysed with 20 mM Tris-HCl (pH 7.4), containing 150 mM NaCl, 0.5% Triton X-100, 1 mM Na_3_VO_4_, 150 mM NaF, 1 mM phenylmethylsulfonyl fluoride, and a protease inhibitor mixture (Sigma-Aldrich, Madrid, Spain) at 4 °C. Homogenates were then clarified by centrifugation at 13,000× *g* for 10 min. Protein from the supernatants was quantified according to Bradford [53], and 100 µg of protein was analyzed by immunoblot using an antibody specific for the phosphorylated form of cPLA_2_α at Ser505 (Cell Signaling, Danvers, MA, USA) [54,55]. The detection of immunoreactive bands was conducted by chemiluminescence (ECLTM, Amersham Biosciences, Little Chalfont, UK).

## 3. Results

**Figure 1:**
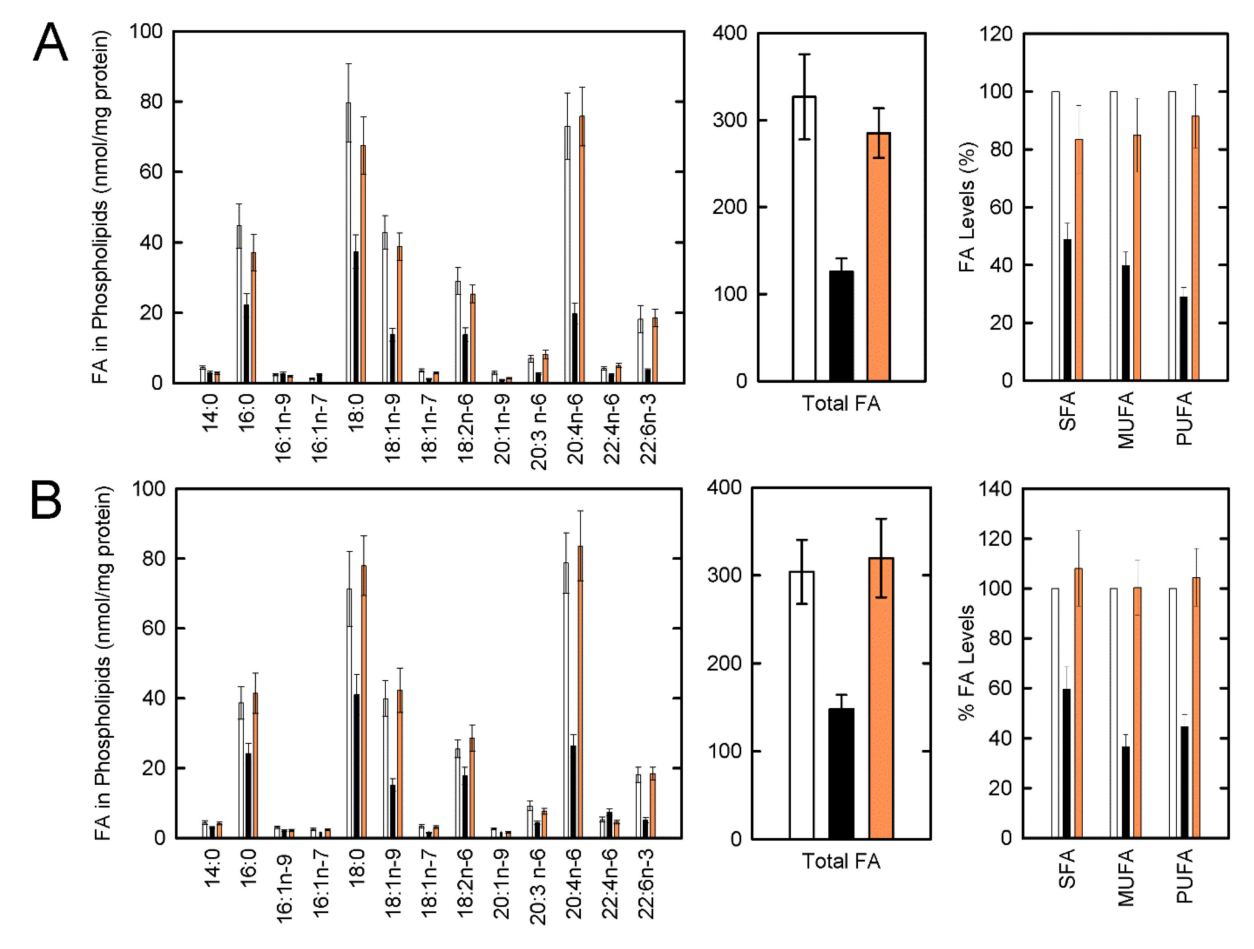
Phospholipid fatty acid content of human monocytes. The cells were either untreated (open bars) or treated with Asp-49 Ca^2+^-dependent secreted phospholipase A_2_ (sPLA_2_) (MT-III) (black bars) or Lys-49 sPLA2 (MT-II) (orange bars) for 1 h (**A**) or 6 h (**B**). Afterward, phospholipids were isolated and their fatty acid content was measured by GC/MS. The profile of fatty acids, the total phospholipid fatty acid amount, and distribution according to the number of double bonds is given. Fatty acids are designated by their number of carbon atoms and, after a colon, their number of double bonds. To differentiate isomers, the *n*−x (*n* minus x) nomenclature is used, where *n* is the number of carbons of a given fatty acid, and x is an integer which, subtracted from *n*, gives the position of the last double bond of the molecule. The data are expressed as mean values ± standard error of three independent determinations. Fatty acid (FA); saturated fatty acid (SFA); monounsaturated fatty acid (MUFA); polyunsaturated fatty acid (PUFA).

The two group IIA sPLA_2_s utilized in this study are from *Bothrops asper* venom and differ in a natural mutation at position 49. Asp49-sPLA_2_, with catalytic activity, was named MT-III; Lys-49-sPLA_2_, devoid of catalytic activity, was named MT-II [34,35,36,37,38]. When added to human monocytes, catalytically active MT-III, at concentrations not compromising cell viability (0.4 µg/mL), promoted an extensive loss of phospholipid-bound fatty acids, as measured by GC/MS (Figure 1).

**Figure 2:**
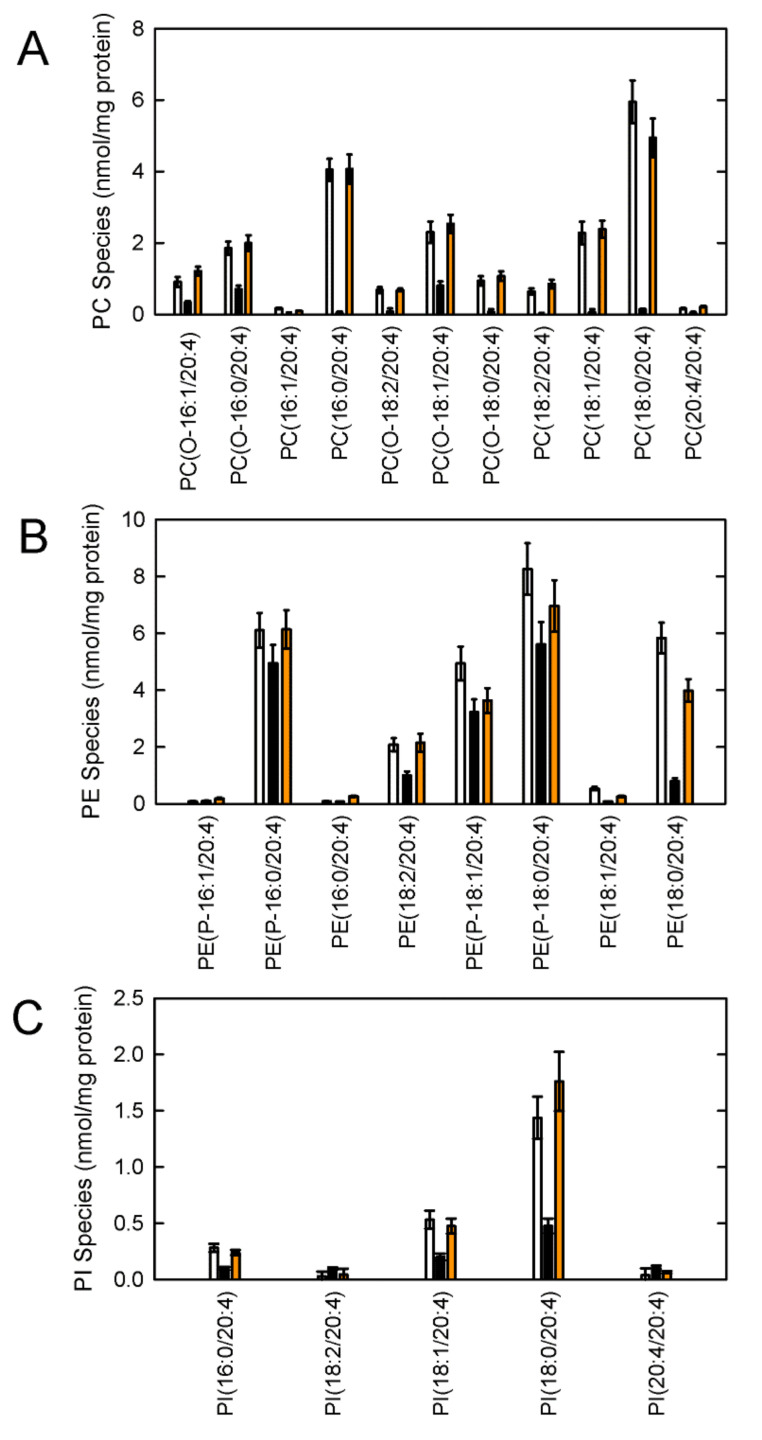
Arachidonic acid (AA)-containing phospholipid species of human monocytes. The cells were either untreated (open bars) or treated with MT-III (black bars) or MT-II (orange bars) for 1 h. Afterward, the distribution profile of AA between choline-containing phospholipids (PC) (**A**), ethanolamine-containing phospholipids (PE) (**B**), and phosphatidylinositol (PI) (**C**) was determined by LC/MS. Fatty chains within the different phospholipid species are designated by their numbers of carbons and double bonds. A designation of O- before the first fatty chain indicates that the sn-1 position is ether linked, whereas a *p*- designation indicates a plasmalogen form (sn-1 vinyl ether linkage). The data are expressed as mean values ± standard error of three independent determinations.

Despite the relatively high phospholipid hydrolysis rates detected in these experiments, cell viability always remained above 90%, as assessed by the MTT assay [56,57,58]. The action of MT-III was prominent on all kinds of fatty acids, including saturated, monounsaturated, and polyunsaturated. Importantly, no significant differences were observed between unstimulated control cells and MT-II-treated cells at any time tested. Since there was little difference in phospholipid hydrolysis between 1 h and 6 h, a 1 h time point was chosen to be employed in all subsequent experiments. The marked decrease in cellular AA (20:4n−6) levels after treating the monocytes with MT-III, as shown in Figure 1, was striking. Given the key role of AA in inflammatory reactions as a precursor of eicosanoids, we set out to characterize further the effect of MT-III on this particular fatty acid. Figure 2 shows the profile of AA-containing glycerophospholipid species of human monocytes, as measured by LC/MS.

Treatment of the monocytes with MT-III, but not MT-II, resulted in a marked decrease in the total cellular content of AA-containing PC and AA-containing PI (Figure 2). Note that some of the most abundant species such as the diacyl species PC(18:0/20:4) or PC(18:1/20:4) almost disappeared after treating the cells with MT-III. Regarding PE species, it was noted that the diacyl species also experienced dramatic decreases, similar to their PC and PI counterparts; however, the plasmalogen forms were much less affected (Figure 2). Although this could indicate that plasmalogen species may not be within the reach of MT-III, it seems likely that, immediately after hydrolysis, these species were rapidly replenished with AA via CoA-dependent transacylation reactions at the expense of diacyl PC species [21,25,26,52,59,60,61].

**Figure 3:**
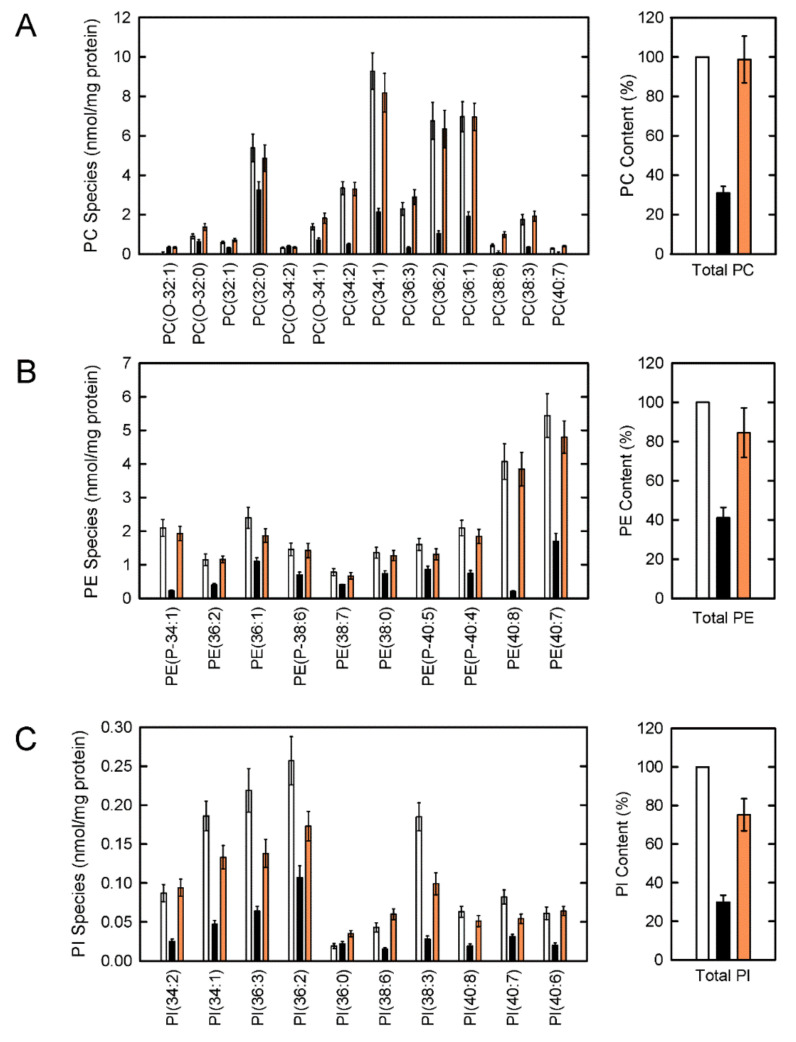
Phospholipid species not containing AA of human monocytes. The cells were either untreated (open bars) or treated with MT-III (black bars) or MT-II (orange bars) for 1 h. Afterward, the cellular content of PC (**A**), PE (**B**), and PI (**C**) molecular species was determined by LC/MS. Species are given in abbreviated form, indicating phospholipid class and number of carbon atoms and double bonds of the two lateral chains together. The data are expressed as mean values ± standard error of three independent determinations.

Figure 3 shows the LC/MS analysis of major phospholipid species not containing AA. In many cases, fragmentation of the *m*/*z* peaks detected in MS analyses yielded fragments corresponding to several species, which made it not possible to unequivocally assign structures to these *m*/*z* peaks. Thus, the data are given in abbreviated form, indicating phospholipid class and number of carbon atoms and double bonds of the two lateral chains together. In Table S1, the fatty acid combinations detected for each *m*/*z* are shown. For example, PI (34:1) represents a mix of PI (18:0/16:1) plus PI (16:0/18:1), and PI (36:2) represents a mix of PI (18:0/18:2) and PI (18:1/18:1).

**Figure 4:**
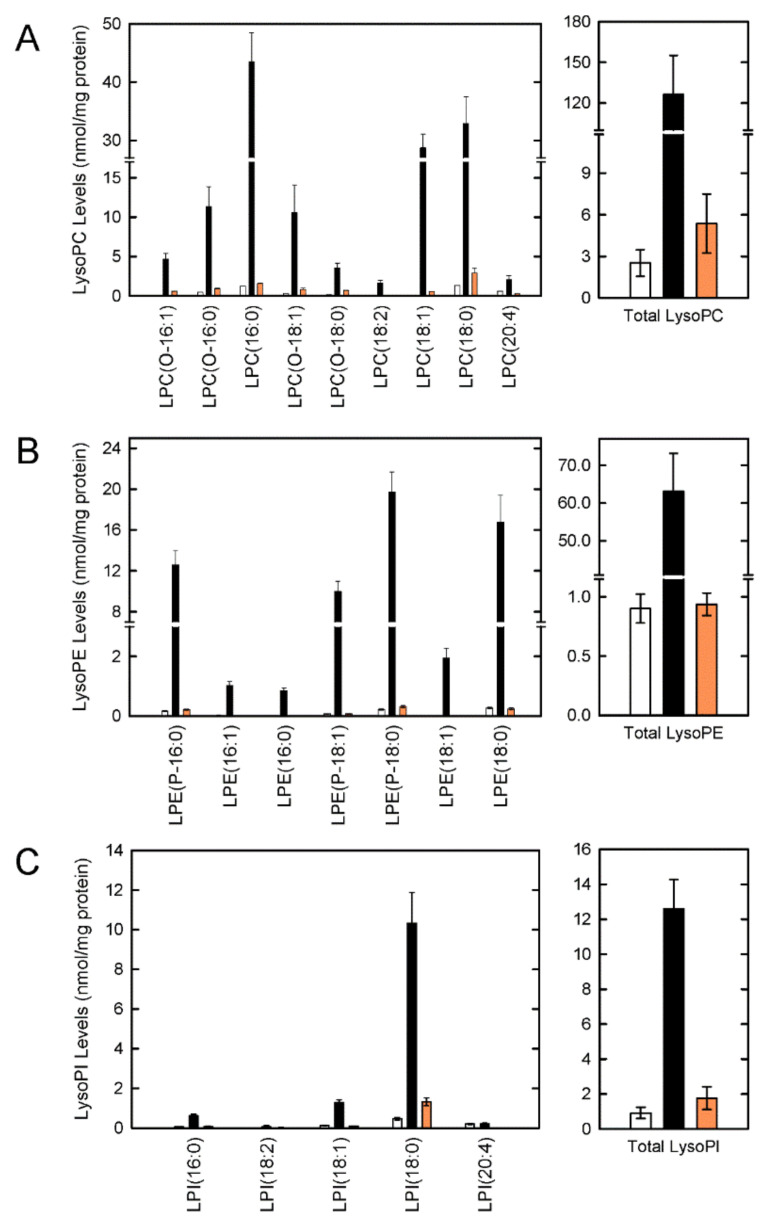
Lysophospholipid molecular species of human monocytes. The cells were either untreated (open bars) or treated with MT-III (black bars) or MT-II (orange bars) for 1 h. The cellular content of lysoPC (**A**), lysoPE (**B**), and lysoPI (**C**) molecular species was determined by LC/MS. Fatty chains within the different lysophospholipid species are designated by their numbers of carbons and double bonds. A designation of O- before the fatty chain indicates an sn-1 ether linkage, whereas a P- designation indicates an sn-1 vinyl ether linkage. The data are expressed as mean values ± standard error of three independent determinations.

Marked reductions in all phospholipid classes were observed in the MT-III-treated monocytes. PE plasmalogen species not containing AA were hydrolyzed to much more extent that their AA-containing counterparts (*cf*. Figure 2B and Figure 3B). Notably, some PI species such as PI (34:1), PI (36:3), PI (36:2) and PI (38:3) showed clear decreases also on stimulation with MT-II (Figure 4C). This finding represents the only positive effect observed in this study for MT-II with regard to lipid turnover. Its significance is unclear at this time but we speculate that it could constitute a ligand-like effect of MT-II related to activation of PI-dependent signaling (e.g., PI 3-kinase or intracellular Ca^2+^-mediated pathways) via ligand binding to elements of the cell surface [31].

To complete the overall lipidomic picture of changes occurring via phospholipid deacylation in the human monocytes, the measurement of lysophospholipid species was also carried out, and the results are shown in Figure 4. We detected a large lysophospholipid production after cellular treatment with MT-III, but not with MT-II, as measured by LC/MS. Consistent with the phospholipid composition of human monocytes [62], lysoPC species were detected in greater abundance, followed by lysoPE and lysoPI.

**Figure 5:**
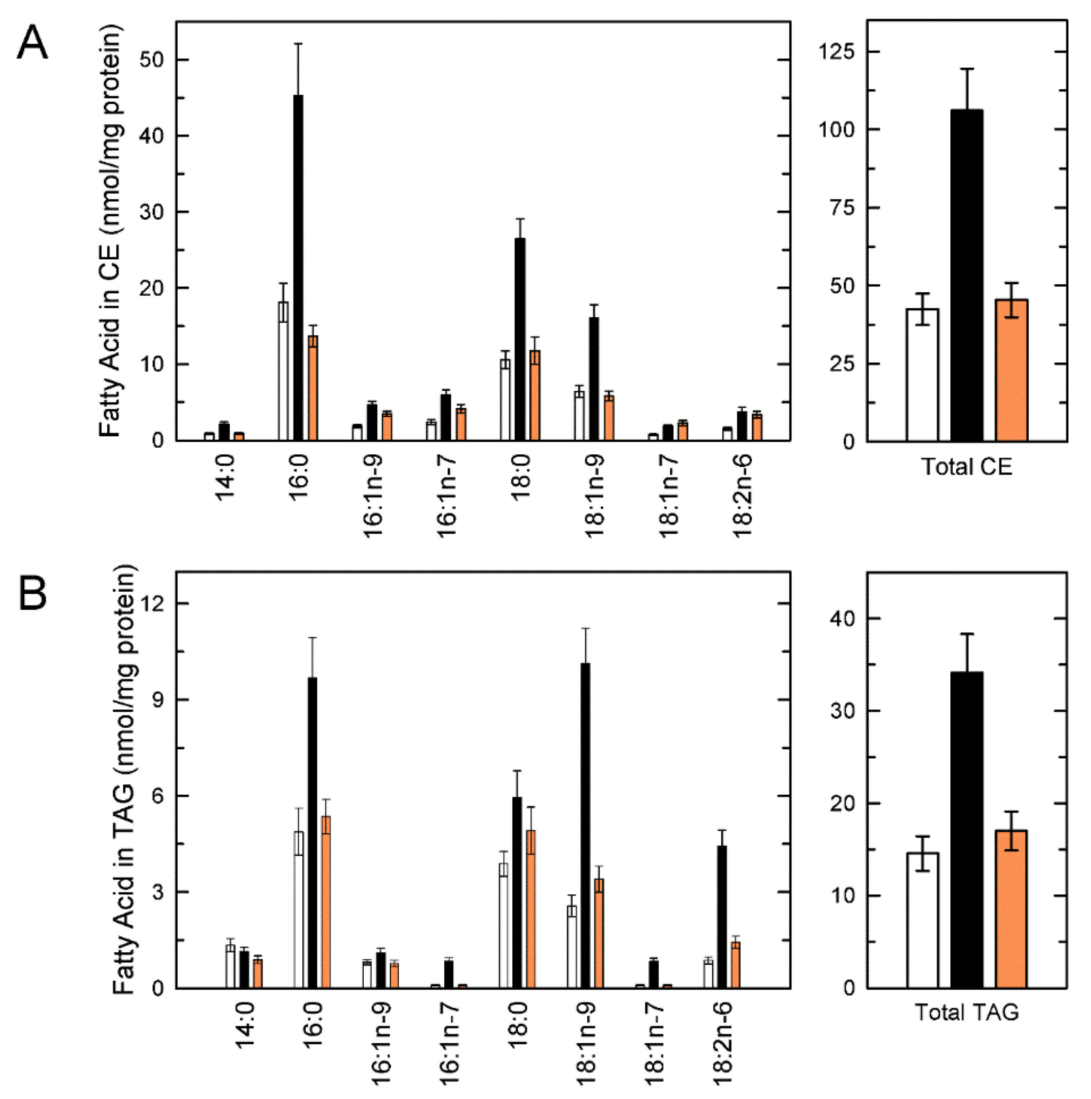
Fatty acid content of neutral lipids in human monocytes. The cells were either untreated (open bars) or treated with MT-III (black bars) or MT-II (orange bars) for 1 h. Afterward, cholesterol esters (CE) (**A**) and triacylglycerol (TAG) (**B**) fractions were isolated and their fatty acid content was measured by GC/MS. The profile of fatty acids, and the total CE or TAG amount are given. The data are expressed as mean values ± standard error of three independent determinations.

In the next series of experiments, we assessed the metabolic fate of the free fatty acids produced upon MT-III treatment. The data shown in Figure 5 indicate that a very significant incorporation of fatty acids did occur into neutral lipid classes, i.e., cholesterol esters and triacylglycerol. Consistent with the results of Figure 1, four of the five the major fatty acids lost from phospholipids—palmitic acid (16:0), stearic acid (18:0), oleic acid (18:1n−9) and linoleic acid (18:2n−6)—were also those that were incorporated in the highest proportion in neutral lipids. Very noticeably, however, the incorporation of AA into neutral lipids was negligible, suggesting other specific metabolic fates for this particular fatty acid.

**Figure 6:**
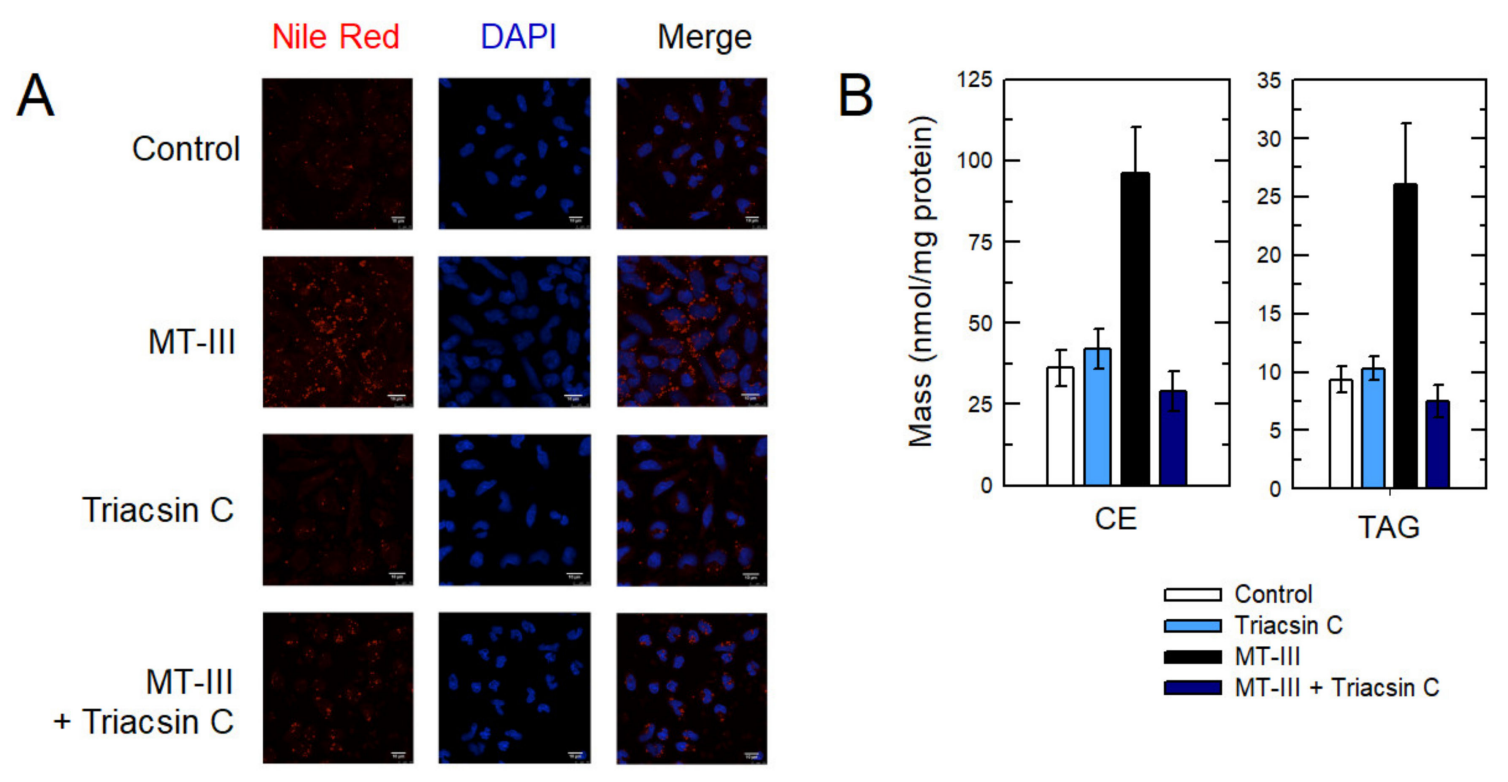
Lipid droplet formation in human monocytes. The cells, pretreated without or with triacsin, were exposed to MT-III as indicated. (**A**) After fixation, cells were stained with Nile Red to visualize lipid droplets (red; left panels) and DAPI to mark the nuclei (blue; central panels). Right panels show the merge. (**B**) Total fatty acid content in cholesterol esters (CE) and triacylglycerol (TAG) was analyzed in cells pretreated without (open bars and black bars) or with (light blue bars and dark blue bars) 3 µM triacsin C, and exposed to MT-III (black bars and dark blue bars) or left otherwise untreated (open bars and light blue bars). The data are expressed as mean values ± standard error of three independent determinations.

To investigate whether the increased synthesis of neutral lipids in the MT-III-treated cells resulted in the formation of lipid droplets, experiments were carried out to visualize these cytoplasmic organelles. Unlike human macrophages, resting human monocytes contain few lipid droplets [46]. Treatment of the cells with MT-III induced a very significant increase in the number of lipid droplets in comparison with control cells, incubated with culture medium alone (Figure 6). Mammalian cells contain five long-chain acyl-CoA synthetases, termed ACSL-1, -3, -4, -5, and -6, and human monocytes express all five of them [46]. The presence of triacsin C, a general inhibitor of long-chain acyl-CoA synthetases [63,64], quantitatively inhibited lipid droplet formation and fatty acid incorporation into neutral lipids (Figure 6). Collectively, these results suggest that lipid droplet production in MT-III-treated monocytes occurs as a consequence of increased availability of intracellular free fatty acids, which are converted into acyl-CoAs, acylated into neutral lipids, and stored in lipid droplets.

**Figure 7:**
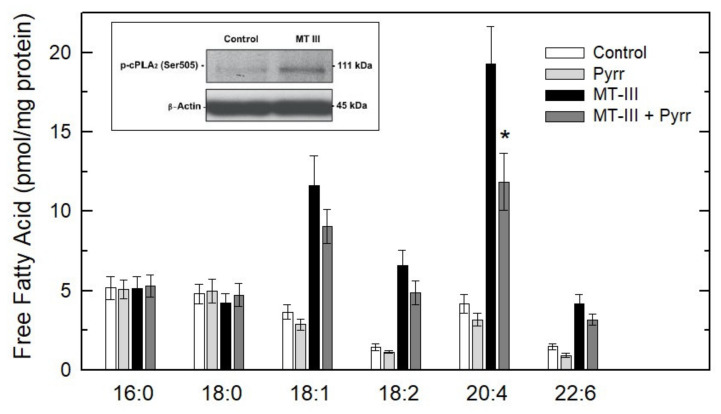
Free fatty acid release by human monocytes. The cells, treated without (open bars and black bars) or with (light gray bars and dark gray bars) 1 μM pyrrophenone (pyrr), were exposed to MT-III (black bars and dark gray bars) or left otherwise untreated (open bars and light gray bars). Free fatty acids were isolated and analyzed by LC/MS. The data are expressed as mean values ± standard error of three individual replicates. * *p* < 0.05, significantly different from cells not treated with pyrrophenone (Student’s t-test). Inset: cell protein was separated by SDS-PAGE and the phosphorylation of cPLA_2_α at Ser^505^ was analyzed by immunoblot using a specific antibody. β-actin was used as a load control.

The absence of incorporation of AA into neutral lipids was unexpected, and prompted us to determine free fatty acid levels in the MT-III-treated cells. The data demonstrated the abundant presence of free AA as well as palmitic, stearic and oleic acids. Lower levels of the polyunsaturated fatty acids linoleic acid and docosahexaenoic acid were also detected (Figure 7). Importantly, only free AA levels were significantly blunted when the analyses were conducted with cells that had been pretreated with pyrrophenone prior to MT-III exposure. Pyrrophenone is a well-established inhibitor of intracellular cytosolic group IVA phospholipase A_2_α (cPLA_2_α), and exhibits more than 1000-fold selectivity for the inhibition of cPLA_2_α versus other types of PLA_2_s, including the group IIA enzymes such as MT-III [65,66,67,68]. There was a tendency for other fatty acids—e.g., oleic acid and linoleic acid—to also decrease after pyrrophenone treatment; however, the differences failed to reach statistical significance. Collectively, these data suggest that MT-III activates cPLA_2_α; therefore, AA mobilization under these conditions would be a composite of the actions of both MT-III and cPLA_2_α. Cross-talk between cPLA_2_α and sPLA_2_ in AA release has often been described during the activation of innate immune cells [14,15,16,17,18,19,20,69,70,71,72,73,74,75,76,77]. Supporting our view that MT-III activates cPLA_2_α, MT-III-treated cells showed increased phosphorylation of cPLA_2_α at Ser505, a hallmark of cPLA_2_α activation [12,13] (Figure 7, inset).

**Figure 8:**
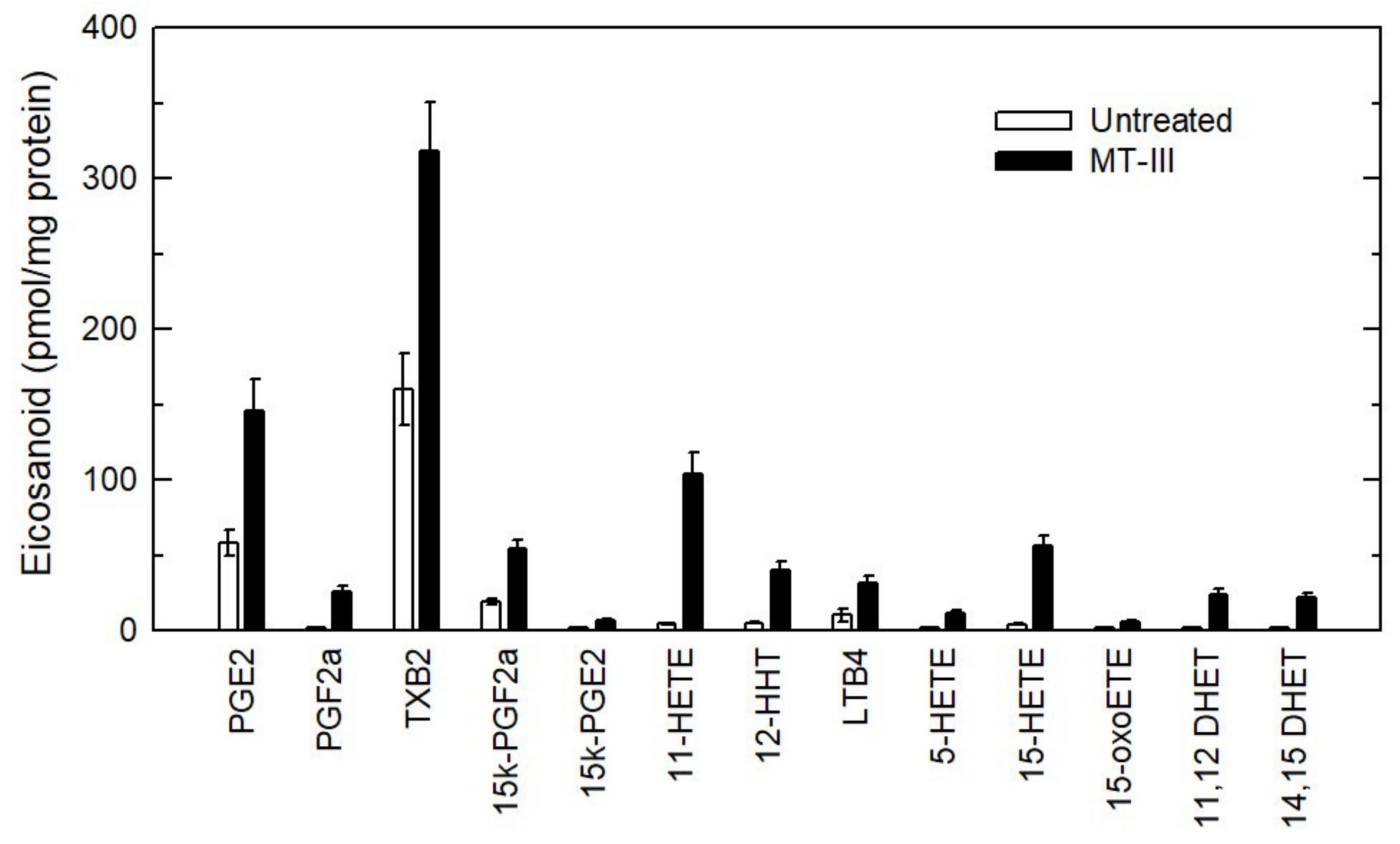
Eicosanoid production by stimulated monocytes. The cells were either untreated (open bars) or treated with MT-III (black bars) for 1h. Afterward, the eicosanoid content in the supernatants was analyzed by LC/MS. The data are expressed as mean values ± standard error of three individual replicates. Prostaglandin E_2_ (PGE2); prostaglandin F_2α_ (PGF2a); thromboxane B_2_ (TXB2); 15-ketoprostaglandin F_2α_ (15k-PGF2a); 15-ketoprostaglandin E_2_ (15k-PGE2); 11-hydroxyeicosatetraenoic acid (11-HETE); 12- hydroxyheptadecatrienoic acid (12-HHT); leukotriene B_4_ (LTB4); 5-hydroxyeicosatetraenoic acid (5-HETE); 15-hydroxyeicosatetraenoic acid (15-HETE); 15-oxoeicosatetraenoic acid (15-oxoETE); 11,12-dihydroxyeicosatrienoic acid (11,12-DHET); 14,15-dihydroxyeicosatrienoic acid (14,15-DHET).

Figure 8 shows that a substantial part of the AA lost from phospholipids by the action of MT-III was metabolized to a variety of eicosanoids, mostly from the cyclooxygenase pathway. Prostaglandin E_2_ and thromboxane B_2_ were the major metabolites produced by the monocytes, in agreement with previous estimates [78,79,80]. Lower amounts of products of the lipoxygenase and cytochrome P450 pathways were also detected upon MT-III stimulation (Figure 8).

## 4. Discussion

This work provides a mass spectrometry-based lipidomic analysis of the actions of group IIA secreted phospholipase A_2_ on human peripheral blood monocytes. Although previous work has dealt with the interactions of this class of enzymes with cell surface structures and subsequent signaling [3,31], no reports, to the best of our knowledge, have characterized the global changes in the cellular lipidome as done in this study.

Group IIA sPLA_2_ is synthesized and secreted by a variety of cells in response to inflammatory cytokines, and is found at large amounts in fluids from inflammatory exudates. Furthermore, group IIA sPLA_2_ enzymes are also present in the venom of scorpions, wasps and, more abundantly, snake venoms, where they behave as relevant inducers of acute inflammation reactions [81]. However, it remains to be clarified how this secreted protein acts on the outer surface of the plasma membrane of mammalian cells to activate immune cells and trigger inflammation. To shed light on this issue, we first analyzed the complete lipidomic profile of metabolites produced by the action of the enzyme. Importantly, AA, a major player in inflammation reactions, is the fatty acid showing the largest decrease in monocyte membranes after MT III exposure, followed by palmitic acid, stearic acid and oleic acid. Other polyunsaturated fatty acids such as linoleic acid, dihomo-γ-linoleic acid and docosahexaenoic acid are also released in smaller quantities and do not contribute significantly to the pool of bioactive oxygenated metabolites produced under these conditions.

We observed that several PC species disappear almost entirely from the membrane. Intriguingly, it has been suggested that membranes enriched in PC may behave as poor substrates for sPLA_2_-IIA [32]. Several studies also indicated that the phospholipid preference may be partially explained by the number of positively charged amino acids and the lack of tryptophan in the interfacial binding site. These residues may constitute key structural determinants that permit binding and hydrolysis to whole membranes [3]. Other sPLA_2_ family members such as the -IB and -V proteins possess tryptophan residues on their putative interfacial binding surfaces; therefore, they show an enhanced capacity to bind to PC-rich vesicles [82]. Our results, using a pathophysiologically relevant setting, raise the concept that in addition to sequence differences, the molecular composition of the membrane to which the sPLA_2_ binds—including protein components—may influence the subsequent hydrolytic steps. Our results may also provide an appropriate experimental frame to relate the catalytic activity of various sPLA_2_ on different classes of phospholipids with their sequences for future studies.

Lipid droplet biogenesis has been demonstrated to be associated with signaling events triggered by inflammation and metabolic stress [83,84,85,86,87]. We show here that the catalytic activity of group IIA sPLA_2_ is required for lipid droplet formation to occur, it probably being the only factor involved, since inactive MT-II does not reproduce the effect. Our results suggest that the extensive hydrolysis of membrane phospholipids promoted by MT-III generates free fatty acids that are channeled to neutral lipids and the formation of cytoplasmic lipid droplets. In support of this view, neutral lipid formation is strongly blunted by the acyl-CoA synthetase inhibitor triacsin C, indicating that the activation of the carboxyl group of a free fatty acid is a required event. In turn, this implicates the participation of CoA-dependent acyltransferase reactions utilizing free fatty acids, not the direct transfer of fatty acids between lipids via CoA-independent transacylation reactions.

A striking feature of the present work is that, of all major fatty acids released by MT-III, AA was excluded from incorporating into neutral lipids. We have recently shown that human monocytes exposed to micromolar amounts of AA do incorporate the fatty acid into neutral lipids, implying that this pathway is fully functional in these cells [88]. This is an interesting concept because recent work has suggested a link between lipid droplets and AA metabolism in mast cells and neutrophils [89,90]. These studies showed that AA recently incorporated into neutral lipids of lipid droplets may be mobilized under activation conditions, thus providing an alternative source of free fatty acid. Therefore, our finding that AA does not incorporate into neutral lipids during exposure of the monocytes to sPLA_2_ clearly suggests that the pathway described in mast cells and neutrophils is not operative, and the fatty acid is used to fulfill other important cellular functions. The most immediate is the direct channeling of AA to the production of proinflammatory eicosanoids that help establish a strong inflammatory reaction. Consistent with this view, we have detected abundant production of proinflammatory mediators, especially those arising from the cyclooxygenase pathway. We did not detect significant formation of putatively anti-inflammatory eicosanoids such as lipoxins or *n*−3 fatty acid derivatives.

In previous work we showed that lipid droplet formation by cells exposed to various stimulants, including MT-III, is blunted by the cPLA_2_α selective inhibitor pyrrophenone [46,58,91]. In this work, we show that pyrrophenone significantly inhibits the accumulation of free AA in the supernatants of MT-III-treated cells. Moreover, MT-III-treated cells demonstrate increased phosphorylation of cPLA_2_α at Ser505. Collectively, the data are suggestive of the possibility that crosstalk exists between cPLA_2_α and MT-IIII. As a matter of fact, evidence has accumulated to suggest that the high AA specificity of cPLA_2_α and the lack of fatty acid selectivity in sPLA_2_s can be combined to achieve specific cellular responses [1,3,14]. Since MT-III causes extensive phospholipid hydrolysis, we speculate that the ensuing membrane disruption may favor Ca^2+^ fluxes that activate intracellular enzymes such as cPLA_2_α. A scenario such as this has even been proposed for catalytically inactive sPLA_2_s, acting via receptor-like mechanisms [3,31]. However, in our studies, inactive MT-II does not induce phospholipid hydrolysis; thus, cPLA_2_α has no active participation in the signaling mediated by this sPLA_2_.

**Scheme 1:**
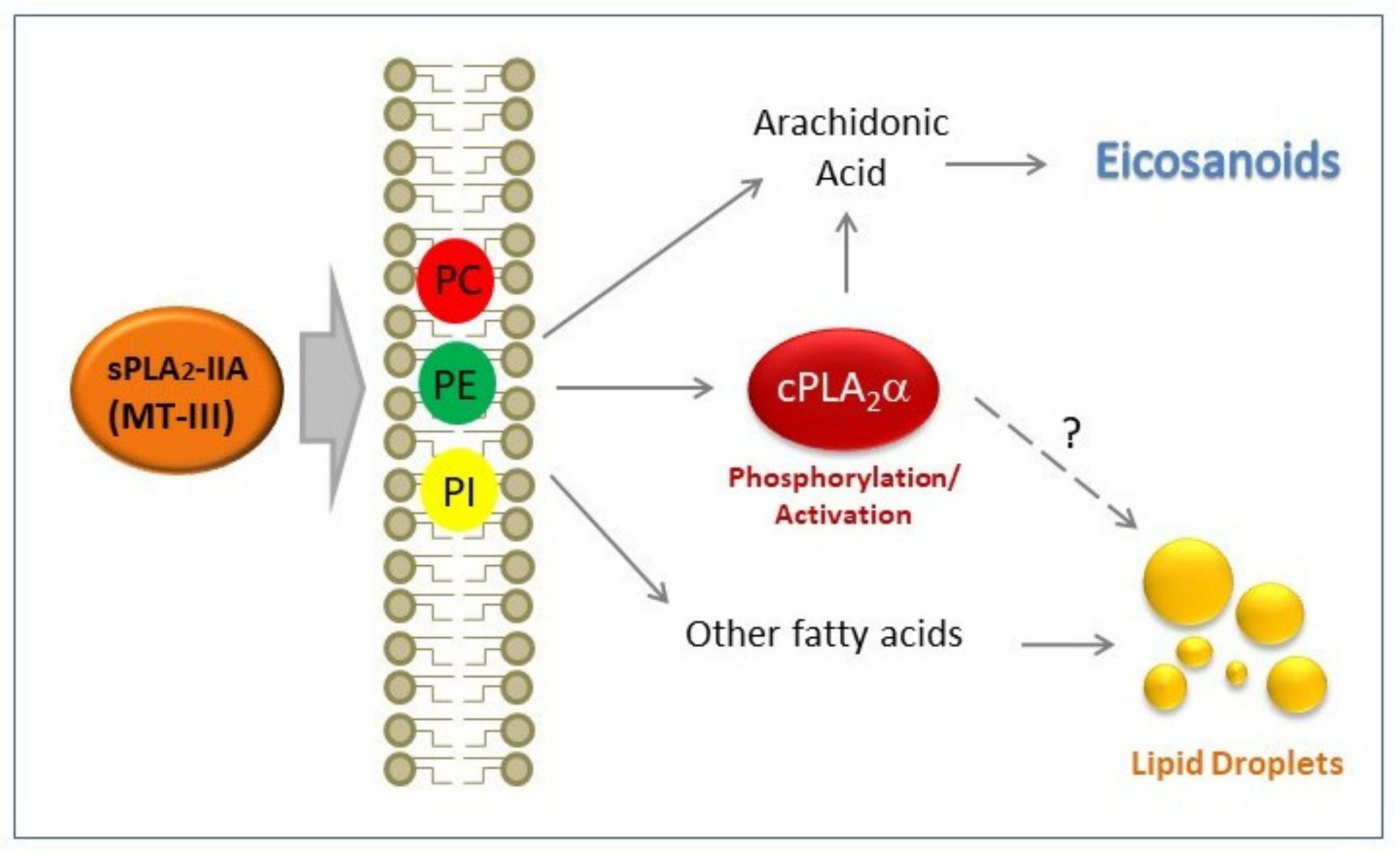
Lipid mediators and cellular responses triggered by catalytically active group IIA sPLA_2_ (MT-III) on human monocytes.

Collectively, our results highlight important actions of catalytically active group IIA sPLA_2_ on the surface of innate immune cells. These actions may trigger different cellular responses, depending on the lipid mediator released (Scheme 1). Clearly, further research will be needed to define the role of MT-III in supplying AA for eicosanoid biosynthesis, the mechanism of crosstalk between MT-III and intracellular cPLA_2_α, and a possible role for the latter in regulating lipid droplet biogenesis, as suggested elsewhere [87,91,92].

## 5. Conclusions

A complex network of chemical mediators including cytokines or eicosanoids characterizes the inflammation process triggered in many diseases or envenomations, involving the hydrolytic action of sPLA_2_s. Regardless of their catalytic activity, it has been demonstrated that PLA_2_s isolated from snake venoms (myotoxins) induce a marked local inflammatory reaction. This is characterized by an early increase in plasma extravasation, edema and a conspicuous infiltration of leukocytes followed by hyperalgesia. All these processes are the result of local and/or systemic concerted action of cytokines such as interleukin-1β, interleukin-6, tumor necrosis factor-α or interferon-γ. Despite this, lipid profiling of the changes induced by these sPLA_2_s on circulating blood cells had not been documented. This study provides an in depth lipidomic profiling of the monocyte response to the direct action of a group IIA sPLA_2_, i.e., MT-III. The data reveal significant connections among lipid droplets biogenesis, cellular signaling, and biochemical pathways that contribute to initiating the inflammatory response.

## Supplementary Materials

The following are available online at https://www.mdpi.com/2218-273X/10/6/891/s1, Table S1: Fatty acid compositions of phospholipid species not containing AA in human monocytes.

## Abbreviations

AA	arachidonic acid
CE	cholesterol esters
cPLA_2_α	group IVA cytosolic phospholipase A_2_α
GC/MS	gas chromatography coupled to mass spectrometry
LC/MS	liquid chromatography coupled to mass spectrometry
PC	choline-containing phospholipids
PE	ethanolamine-containing phospholipids
PI	phosphatidylinositol
sPLA_2_	secreted phospholipase A_2_
TAG	triacylglycerol
