# Supplementary material for: A Lipidomic Perspective of the Action of Group IIA Secreted Phospholipase A2 on Human Monocytes: Lipid Droplet Biogenesis and Activation of Cytosolic Phospholipase A2α
Source: Biomolecules. 2020 Jun 10;10(6):891. doi: 10.3390/biom10060891 (PMC7355433; doi:10.3390/biom10060891)
Supplement: Supplementary file 1 [file biomolecules-10-00891-s001.pdf]

TABLE S1 – Fatty acid compositions of phospholipid species not containing AA in human monocytes

**Phospholipid**[1]	**m/z**[2]	**Molecular species**[3]
PC(O-32:1)	718.5	PC(O-16:0,16:1), PC(O-18:1,14:0)
PC(O-32:0)	720.5	PC(O-16:0,16:0), PC(O-18:0,14:0)
PC(32:1)	732.5	PC(14:0,18:1), PC(16:0, 16:1)
PC(32:0)	734.5	PC(14:0,18:0), PC(16:0,16:0)
PC(O-34:2)	744.5	PC(O-16:0,18:2), PC(O-18:1,16:1)
PC(O-34:1)	746.5	PC(O-16:0,18:1), PC(O-18:0,16:1), PC(O-18:1,16:0)
PC(34:2)	758.5	PC(16:0,18:2), PC(16:1,18:1)
PC(34:1)	760.5	PC(16:0,18:1), PC(16:1,18:0)
PC(36:3)	784.5	PC(16:0,20:3), PC(18:0,18:3), PC(18:1,18:2)
PC(36:2)	786.5	PC(18:0,18:2), PC(18:1,18:1)
PC(36:1)	788.5	PC(16:1,20:0), PC(18:0,18:1)
PC(38:6)	806.5	PC(16:0, 22:6), PC(16:1,22:5), PC(18:1,20:5), PC(18:3,20:3)
PC(38:3)	812.5	PC(18:0,20:3), PC(18:3, 20:0)
PC(40:7)	832.5	PC(18:1,22:6), PC(18:2,22:5), PC(18:3,22:4)
PE(P-34:1)	700.5	PE(P-16:0,18:1), PE(P-18:0,16:1), PE(P-18:1,16:0)
PE(36:2)	742.5	PE(18:0, 18:2), PE(18:1,18:1)
PE(36:1)	744.5	PE(16:1,20:0), PE(18:0,18:1)
PE(P-38:6)	746.5	PE(P-16:0,22:6), PE(P-16:1,22:5), PE(P-18:1,20:5)
PE(38:7)	760.5	PE(16:1,22:6), PE(18:2,20:5)
PE(38:0)	774.5	PE(18:0,20:0)
PE(P-40:5)	776.5	PE(P-18:0,22:5), PE(P-18:1,22:4)
PE(P-40:4)	778.5	PE(P-18:0,22:4), PE(P-18:1,22:3)
PE(40:8)	786.5	PE(18:2,22:6), PE(18:3,22:5)
PE(40:7)	788.5	PE(18:1,22:6), PE(18:2,22:5), PE(18:3,22:4)
PI(34:2)	833.5	PI(16:0,18:2), PI(16:1,18:1)
PI(34:1)	835.5	PI(16:0,18:1), PI(16:1,18:0)
PI(36:3)	859.5	PI(16:0,20:3), PI(18:0,18:3), PI(18:1,18:2)
PI(36:2)	861.5	PI(18:0,18:2), PI(18:1,18:1)
PI(36:0)	865.5	PI(16:0,20:0), PI(18:0,18:0)
PI(38:6)	881.5	PI(16:0, 22:6), PI(16:1,22:5), PI(18:1,20:5)
PI(38:3)	887.5	PI(18:0,20:3), PI(18:3, 20:0)
PI(40:8)	905.5	PI(18:2,22:6), PI(18:3,22:5), PI(20:3,20:5)
PI(40:7)	907.5	PI(18:1,22:6), PI(18:2,22:5), PI(18:3,22:4)
PI(40:6)	909.5	PI(18:0,22:6), PI(18:1,22:5), PI(18:2,22:4)

[1]Major phospholipids not containing AA, detected by LC/MS, are given in abbreviated form (phospholipid class and number of carbon atoms and double bonds of the two lateral chains together).

[2]$[M+H]^+$ for PC; $[M-H]^-$ for PE and PI.

[3]Molecular species were deduced from the fatty acid fragments detected in MS^2 (PE and PI species) or MS^3 (PC species) experiments.
